# Kynurenine and oxidative stress in children having learning disorder with and without attention deficit hyperactivity disorder: possible role and involvement

**DOI:** 10.1186/s12883-022-02886-w

**Published:** 2022-09-20

**Authors:** Ayman Kilany, Neveen Hassan Nashaat, Hala M. Zeidan, Adel F. Hashish, Mostafa M. El-Saied, Ehab Ragaa Abdelraouf

**Affiliations:** 1 Medical Research and Clinical Studies Institute, Children with Special Needs Research Department, National Research Centre, Elbuhouth Street, Cairo, 12622 Dokki Egypt; 2 Pediatric Neurology Research Field, Medical Research Centre of Excellence, National Research Centre, Cairo, Egypt; 3 Learning Disability and Neurorehabilitation Research Field, Medical Research Centre of Excellence, National Research Centre, Cairo, Egypt

**Keywords:** Learning disorder, ADHD, Kynurenine, Oxidative stress, Cognitive abilities

## Abstract

**Background:**

The etiological and pathophysiological factors of learning disorder (LD) and attention deficit hyperactivity disorder (ADHD) are currently not well understood. These disorders disrupt some cognitive abilities. Identifying biomarkers for these disorders is a cornerstone to their proper management. Kynurenine (KYN) and oxidative stress markers have been reported to influence some cognitive abilities. Therefore, the aim was to measure the level of KYN and some oxidative stress indicators in children with LD with and without ADHD and to investigate their correlations with the abilities of children with LD.

**Methods:**

The study included 154 participants who were divided into 3 groups: one for children who have LD (*N* = 69); another for children with LD and ADHD (*N* = 31); and a group for neurotypical (NT) children (*N* = 54). IQ testing, reading, writing, and other ability performance evaluation was performed for children with LD. Measuring plasma levels of KYN, malondialdehyde, glutathione peroxidase, and superoxide dismutase by enzyme-linked immunosorbent assay was performed for all participants.

**Results:**

Some IQ measures and learning skills differed between the first two groups. The biochemical measures differed between children with LD (with and without ADHD) and NT children (*p* < 0.001). However, the biochemical measures did not show a significant statistical difference between the first two groups. KYN and glutathione peroxidase levels were correlated with one-minute writing and at-risk quotient, respectively (*p* = 0.03;0.04). KYN and malondialdehyde showed the highest sensitivity and specificity values.

**Conclusion:**

These biochemical measures could be involved or have a role in the abilities’ performance of children with specific learning disorder.

## Introduction

Learning disorder (LD) and attention deficit hyperactivity disorder (ADHD) are neurodevelopmental disorders that influence some cognitive abilities [1]. In some previous studies, the comorbidity between these disorders was as high as 60% [2]. The etiological factors of both LD and ADHD are not yet fully understood. Some common hereditary factors between these disorders were found. Furthermore, children with both disorders were found to have executive functions and short-term memory deficits besides reaction time differences [3]. The coexistence of these disorders could suggest shared neurobiological changes. The kynurenine system and oxidative stress have been linked to cognitive functioning through a variety of complex mechanisms, including changes in neurotransmitter levels, receptor sensitivity, and immune response in the brain [4, 5].

Kynurenine (KYN) is one of the tryptophan metabolites (an essential amino acid), and its pathway activation has been linked to stress exposure and chronic inflammation. KYN is considered neurotoxic and it has been reported to be implicated in central nervous system disorders including Huntington’s disease, Alzheimer’s disease, and depression, all of which compromise memory and fine motor performance, among other problems [6]. Furthermore, the activation of the KYN pathway has been reported to induce oxidative stress [7].

Oxidative stress could lead to reversible or irreversible forms of oxidative modifications of cellular proteins. The modifications have been reported to be an etiological step of cellular dysfunction, particularly neurons and other brain cells. Antioxidants, which are produced by the human body, neutralize oxygen-derived free radicals, or reactive oxygen species. These species indiscriminately attack vital molecules, such as proteins, DNA, carbohydrates, and fats, which are essential structures of every cell. This would alter the function of these molecules and impair the normal course of their metabolism in cells. It’s worth noting that low concentrations of reactive oxygen species serve a useful purpose in the body for regulation of cellular homeostasis. Reactive oxygen species are necessary to activate the pathways that upregulate the activity of various antioxidant enzymes, including glutathione system enzymes. Therefore, oxidative stress could be defined as “a state where oxidative forces exceed the antioxidant systems due to loss of the balance between them”. Oxidative stress has been implicated in the pathogenesis of neurodevelopmental disorders and neurodegenerative disorders, such as vascular cognitive impairment, which hinders memory and learning in adults [8]. In healthy adults, executive skills have been reported to be inversely related to oxidative stress [9]. Oxidative stress can be monitored by several measures, such as lipid peroxidation represented by Malondialdehyde (oxidative force marker), glutathione peroxidase, and super oxide dismutase (antioxidant markers).

Malondialdehyde (MDA) is a lipid peroxidation-related measure. It has long been recognized as a biomarker for oxidative stress. It is one of many reactive electrophile species that produce toxic stress in cells by forming covalent protein adducts. These adducts are known as advanced lipoxidation end products, which cause oxidative damage to cells, including neural cells [10]. On the other hand, human body has proteins that protect the cells from the sequalae of cellular reactions and their toxic products. These proteins include enzymes such as glutathione peroxidase (GPx) and super oxide dismutase (SOD). These enzymes are responsible, among others, for reducing the toxic free oxygen radicals. In some animal studies, they were linked to memory performance and hippocampus functioning [11]. GPx limits hydrogen peroxide damaging effect by reducing it into water, thus modulating the growth factor-mediated signal transduction, maintenance of normal thiol redox-balance and mitochondrial function which all are essential for proper neuronal metabolism [12]. SOD catalyzes the conversion of oxide and hydrogen peroxide to non-toxic compounds which reduces oxidative stress and participates in improving cell signaling. Moreover, SOD plays a critical role in inhibiting oxidative inactivation of nitric oxide. It prevents peroxynitrite formation and, consequently, reduces endothelial damage and mitochondrial dysfunction [13].

All of the previous biochemical measures were implicated in memory and learning in animal studies, and a few human adult studies which targeted neurological disorders influencing memory, cognition, and motor abilities [14, 15]. Investigating such measures in children with LD and ADHD could reveal possible biochemical abnormalities in these children, as well as provide insight into their likely association with these disorders. The goal of this study was to compare the plasma levels of KYN, MDA, GPx, and SOD in children with LD with and without ADHD to those in neurotypical (NT) children to understand if there was any difference in these levels and if there was any correlation with their cognitive and learning abilities. The diagnostic value of these biochemical measures was also investigated.

## Methods

### Participants and procedures

The participants (a total of 154) in this cross-sectional study, which followed the checklist for STROBE,^1^ were divided into three groups: children with LD, children with LD and ADHD, and NT children. Children were diagnosed as LD with or without coexisting ADHD (LD ± ADHD) according to the criteria of the diagnostic and statistical manual of mental disorders, fifth edition [1]. The participants visited the learning disability and neurorehabilitation research clinic or the pediatric neurology research clinic at the Medical Research Centre of Excellence of the National Research Centre. They were included when their age range was 6–14 years and they were not taking medications. Children with additional neurological signs, neuropsychiatric disorders, hearing impairment, intellectual disability, dysmorphic features suggestive of a syndrome, or a history of motor delay were excluded from the study. Neurotypical children were volunteers who agreed to participate in the study. They were enrolled in the national schooling system, where they excelled academically. They had the same age range and sex distribution as the other groups. Children with a history of motor or language delays were excluded from the study. Written informed consents were obtained from the parents of participants. The study was approved by the medical research ethics committee of the National Research Centre. There were 69 children with LD [47 males, 22 females; age range: 6–13.4 (8.5 ± 1.6)]. There were 31 children with LD and ADHD [20 males, 11 females; age range: 6–11 (8.2 ± 1.4)]. There were 54 NT children [35 males, 19 females; age range: 6–12 (8.5 ± 1.8)]. History taking, clinical examination, Stanford Binet intelligence scale, fifth edition, and dyslexia assessment test were done. EEG was performed for participants with LD ± ADHD to fulfill the exclusion criteria. For those with ADHD, Conners rating scale was done. MRI and audiological evaluation were performed when necessary (e.g., history of perinatal insult, family history of CNS malformations, soft neurological signs, repeated attacks of otitis media with effusion). Stanford Binet intelligence scale, fifth edition estimates total intelligence quotient (IQ), verbal and non-verbal IQ, as well as five subtests: fluid reasoning, knowledge, quantitative reasoning, visuo-spatial abilities and working memory [16, 17]. The dyslexia assessment test has 11 subtests that assess reading, writing, memory, and some linguistic abilities of the children, such as phonological awareness. The final score is called at-risk quotient. It increases as the severity of the learning disorder increases. All the subtests’ raw scores increase with better performance, except rapid-naming subtest, where the scores increase with lower performance. The test was designed for children up to 10 years and 6 months. Considering the lack of a standardized test in Arabic for children older than this age, the two children who were older than this age were subjected to this test, and the standardized tables for the age of 10 years and 6 months were used for obtaining their at-risk quotients. Their scores were even less than what is expected from children who are 10 years and 6 months [18, 19]. For people with ADHD, the Conners rating scale was utilized to determine the severity of their condition [20, 21]. Venous blood samples were obtained from all the included participants. Measures were determined in plasma by the enzyme-linked immunosorbent assay. KYN was measured according to method of Gautam et al. [22]. Plasma MDA was measured as an indicator of lipid peroxidation according to the method described by Chauhan et al. [23]. The GPx measurement was performed according to Jacobson et al. [24]. The SOD measurement was performed according to Afrazeh et al. [25].

### Statistical analysis

The data obtained were collected, tabulated and then analyzed using the Statistical Package for Social Sciences. Descriptive statistics were done for quantitative data as mean ± standard deviation (SD), number and percentage for qualitative data. Inferential analysis was performed for quantitative variables using the independent t-test and for qualitative data using the Chi square test. Correlations between biochemical measures and total IQ, the at-risk quotient, and subtests of the dyslexia assessment test were investigated. For correlation analysis, Spearman correlation coefficient was used. P was considered significant when it was at or less than 0.05.

## Results

### Scores of the Stanford Binet intelligence scale-fifth edition

The IQ range was 70–109 (90.7 ± 9.7) in the LD group. The scores for working memory and non-verbal abilities were the lowest. The scores for quantitative reasoning were the highest. In the group of LD with ADHD, the IQ range was 80–114 (95.4 ± 7.7). The scores for working memory were the lowest. The fluid reasoning scores were the highest. Children with LD and ADHD outperformed children with LD in terms of overall IQ, verbal IQ, fluid reasoning, and knowledge with a significant statistical difference (*p* ≤ 0.05) (Table 1).

**Table 1:** Comparison between the group of children with LD and the group with LD and ADHD regarding the results of the IQ assessment performed by the fifth edition of the Stanford Binet intelligence scale

Scale items	Mean ± SD in LD group	Mean ± SD in LD with ADHD group	*P* value
Total IQ	90.7 ± 10	95.4 ± 12	0.04*
Fluid reasoning	91.5 ± 15	99.1 ± 14	0.01*
Knowledge	91.3 ± 12	96.4 ± 11	0.04*
Quantitative reasoning	94.5 ± 10	98.4 ± 13	0.1
Visuo-spatial	90.6 ± 12	93.1 ± 12	0.3
Working memory	86.3 ± 11	90.5 ± 14	0.1
Non-verbal IQ	88.6 ± 13	93.3 ± 12	0.09
Verbal IQ	91.6 ± 11	96.7 ± 10	0.02*

*IQ* Intelligence Quotient, *LD* Learning disorder, *ADHD* Attention deficit hyperactivity disorder, *SD* Standard deviation

* significant (*p* ≤ 0.05)

### Participants’ performance in the dyslexia assessment test

In children with LD, the at-risk quotient ranged from 0.6 to 2.8 (1.76 ± 0.7). In children with LD and ADHD, the at-risk quotient ranged from 0.3 to 3.1 (1.68 ± 0.6). The majority of participants in both groups manifested deficits in verbal fluency, rapid naming, and one-minute writing. Comparison between the groups concerning percentage of deficits revealed that the ADHD group showed more percentage of deficits in nonsense passage reading, semantic fluency, verbal fluency, backward digit span, postural stability, and bead threading when compared to the LD group. The difference between the groups was significant regarding postural stability, and bead threading only (Table 2).

**Table 2:** Scores and percentage of participants with deficits in the subtests of dyslexia assessment test in the group of children with LD and the group with LD and ADHD

Subtests	Mean ± SD of raw scores in LD group	Percentage of participants with deficits in LD group (approximated %)	Mean ± SD of raw scores in LD with ADHD group	Percentage of participants with deficits in LD with ADHD group (approximated%)	*p*
Rapid naming	105 ± 6	93	96 ± 8	90	0.6
Bead threading	5.5 ± 1.4	11	5.3 ± 1.6	26	0.05*
One minute reading	10.6 ± 11.3	58	13.5 ± 10	58	1
Posture stability	8.8 ± 2.7	16	10.2 ± 2.3	39	0.01*
Phonemic segmentation	4 ± 3.2	75	4.1 ± 3.5	71	0.6
Two-minute spelling	5 ± 4.6	66	7.6 ± 4.2	65	0.9
Backward digit span	3 ± 1.4	37	2.6 ± 1.6	48	0.3
Nonsense passage reading	13 ± 14	66	16 ± 11	74	0.4
One-minute writing	5.1 ± 2.5	93	5.1 ± 2.3	90	0.6
Verbal fluency	2.1 ± 1.3	96	2.7 ± 1.7	97	0.8
Semantic fluency	7.7 ± 2.3	77	7.2 ± 2.1	84	0.4

*LD* Learning disorder, *ADHD* Attention deficit hyperactivity disorder, *SD* Standard deviation

* significant (*p* ≤ 0.05)

### Results of Conner’s rating scale

All of the scale’s items showed above average level except the perfectionism item which showed average score. The details of the scale are presented in Table 3.

**Table 3:** The Conner’s scale Scores of the children with learning disorder and attention deficit hyperactivity disorder

Items	Mean of standard Scores	Standard deviation	Interpretation
Oppositional symptoms	75	12	highly elevated score
Cognitive manifestations	75	8	highly elevated score
Hyperactivity manifestation	77	7	highly elevated score
Anxious-shy behavior	60	15	Mildly elevated score
Perfectionism	55	3	Average score
Social problems	69	15	Very elevated score
Psychosomatic features	65	17	Moderately elevated score
Restless behavior	77	13	highly elevated score
Emotional liability	69	7	Very elevated score

### Comparison between the groups regarding the biochemical measures

For the participants with LD ± ADHD, KYN and MDA levels were higher than NT children, with a significant statistical difference. GPx, and SOD levels were lower in children having LD± ADHD with significant statistical difference (Tables 4, 5). A Comparison between the group of participants with LD and the group of children with LD and ADHD regarding all biochemical measures revealed non-significant statistical difference (Table 6).

**Table 4:** Comparison between the group of children with LD and the group of neurotypical children regarding the biochemical measures

Substance	Mean in LD group	SD	Mean in NT children	SD	t	*p*
kynurenine (ng/ml)	479.7	150.8	274.3	117.8	8.2	< 0.001 *
Malondialdehyde (nmol/ml)	2.09	0.82	0.8	0. 1	11.3	< 0.001 *
glutathione peroxidase (ng/ml)	48.3	17	56.6	12.9	-3	0.002*
Super Oxide Dismutase (μg/ml)	2456.6	1310.5	3795.5	1698.7	-4.9	< 0.001 *

*LD* Learning disorder, *SD* Standard deviation, *NT* Neurotypical

* significant (*p* ≤ 0.05)

**Table 5:** Comparison between the group of children with LD and ADHD and the group of neurotypical children regarding the biochemical measures

Substance	Mean in LD with ADHD group	SD	Mean in NT children	SD	t	*p*
kynurenine (ng/ml)	419.2	157	274.3	117.8	4.8	< 0.0001*
Malondialdehyde (nmol/ml)	1.8	0.8	0.8	0. 1	9.1	< 0.0001*
glutathione peroxidase (ng/ml)	44.6	17.9	56.6	12.9	-3.5	0.0006*
Super Oxide Dismutase (μg/ml)	2564.9	1110.9	3795.5	1698.7	-3.6	0.0005*

*LD* Learning disorder, *ADHD* Attention deficit hyperactivity disorder, *NT* Neurotypical, *SD* Standard deviation

* significant (*p* ≤ 0.05)

**Table 6:** Comparison between the group of children with LD and the group with LD and ADHD regarding the biochemical measures

Measures	Mean in LD group	SD	Mean in LD with ADHD group	SD	t	*p*
kynurenine (ng/ml)	479.7	150.8	419.2	157	1.8	0.07
Malondialdehyde (nmol/ml)	2.09	0.82	1.8	0.8	1.6	0.1
glutathione peroxidase (ng/ml)	48.3	17	44.6	17.9	0.9	0.3
Super Oxide Dismutase (μg/ml)	2456.6	1310.5	2564.9	1110.9	-0.4	0.6

*LD* Learning disorder, *ADHD* Attention deficit hyperactivity disorder, *SD* Standard deviation

### Correlation analysis in the group of children with LD

The level of KYN was found to be inversely correlated with one-minute writing scores, with a significant statistical difference (*r* = -0.2; *p* = 0.03). The GPx level was negatively correlated with the at-risk quotient (*r* = -0.4, *p* = 0.04). This indicated that higher GPx levels were linked to better performance in the tested aptitudes. No other statistically significant correlations were detected.

### Diagnostic utility of biochemical measures

The KYN level (281.50 ng/ml) showed 92% sensitivity and 70% specificity. Furthermore, the MDA level (1.00 nmol/ml) showed 89.9% sensitivity and 89% specificity. These two measures exhibited higher sensitivity and specificity compared to the other measures (Table 7).

**Table 7:** Area under the curve of the biochemical measures in the group of children with learning disorder

Items	Area under the curve	Cut-off value	Sensitivity	Specificity
kynurenine (ng/ml)	0.848	281.50	92%	70%
Malondialdehyde (nmol/ml)	0.948	1.00	89.9%	89%
glutathione peroxidase (ng/ml)	0.654	59.2	75%	56%
Super Oxide Dismutase (μg/ml)	0.726	3670.00	81.2%	70%

## Discussion

The etiological and pathophysiological factors of LD and ADHD are currently not well understood. Therefore, identifying possible biomarkers for these disorders is a cornerstone for better understanding of these disorders and for proper diagnosis, follow-up, and management. This study is the first to investigate such biochemical markers in children with LD ± ADHD.

The ADHD group had higher overall IQ, verbal IQ, fluid reasoning, and knowledge subtest scores than the children who have LD without ADHD. However, the ADHD group exhibited a larger percentage of deficits in certain aptitudes related to reading, memory, and eye-motor coordination, such as nonsense passage reading, backward digit span, semantic fluency, bead threading, and postural stability when compared to the LD group without ADHD. These more frequent deficits in the group with ADHD, despite having a higher IQ level, could be linked to their attention problem considering that the learning tasks require sustained attention. Furthermore, children with ADHD were reported to manifest executive function difficulties [26]. These factors may have contributed to the observed abnormalities in reading and other abilities involved in learning and motor performance.

The biochemical parameters of children with LD ± ADHD differed from those of NT children with a significant statistical difference, indicating abnormalities in the KYN pathway and the existence of oxidative stress in children with LD. The KYN level was noticed to be high in the LD group. The excess KYN level could reflect deficits in its conversion to active brain modulators. The KYN system has been reported to influence the brain by reducing the action of dopamine, acetylcholine, and gamma amino butyric acid [27, 28]. Balanced levels of these neurotransmitters are essential for appropriate brain function and adequate cognitive performance. The integrity of cognitive abilities is fundamental for proper learning, information processing, and executive functions fulfillment [29]. Furthermore, the KYN system has been hypothesized to impact the serotonin/melatonin system in the brain [30]. Children with ADHD were reported to exhibit low levels of serotonin and melatonin. These neurotransmitters were reported to be involved in impulsivity and attention. High KYN levels could have a negative effect on the serotonin and melatonin systems leading to reduction of their levels [31–33]. Moreover, KYN stands in a unique position to represent the effect of environmental factors on cognition and behavior. This system is induced by stress and chronic inflammation, as high cortisol induces the enzymes involved in KYN pathway. This leads to elevation of blood cytokines [34]. Peripheral cytokines can influence the central nervous system via targeting its own immune cells such as microglia, which, in turn, induce central inflammation [5, 30]. This might reduce neuroplasticity and negatively impact the neurotransmitters balance in the brain [35]. Additionally, high KYN level was linked to mitochondrial damage and oxidative stress induction [34]. Children with LD ± ADHD are liable to stress that is induced by their parents and the school system. Chronic stress induces oxidative stress, exaggerating the biochemical imbalance in those children [36]. Oxidative stress, on the other hand, has been shown to increase the kynurenine pathway rather than the normal tryptophan metabolism pathway, increasing the burden of the physiological processes and creating a vicious cycle [7]

Markers for oxidative stress have been targeted in this study. Participants with LD ± ADHD manifested oxidative stress in the form of high MDA, low GPx, and SOD. MDA is one of the final products of polyunsaturated fatty acids peroxidation in cells. Any rise in free radicals leads to overproduction of MDA. The MDA level is a typical indictor of oxidative stress and a lack of antioxidant status. It is one of the many reactive electrophile species that generate toxic stress in cells and form covalent protein adducts called advanced lipoxidation end products, which lead to oxidative damage. Oxidative damage includes oxidative modification of cellular macromolecules, induction of cell death by apoptosis or necrosis, as well as structural tissue damage [37]. Elevated levels of MDA were detected in rats with Alzheimer’s disease who manifested impaired memory and learning. Furthermore, supplementing these rats with a medication that decreases oxidative stress improved their memory and learning [38]. In rats with vascular brain lesions, lower MDA levels were associated with better learning and memory [39]. Furthermore, MDA level was correlated to working and declarative memory types in major depressive disorder, which highlights the impact of this biochemical measure on the human brain [40]. Previous studies which targeted the ADHD population demonstrated contradicting results regarding their oxidative stress status [41, 42]. However, MDA levels in participants with LD ± ADHD in this study were higher than control subjects, with a significant statistical difference (*p* < 0.0001 in both groups). It showed high sensitivity and specificity values. This could offer MDA as a useful biomarker for learning disorder.

The levels of GPx and SOD were reduced in the LD group indicating oxidative stress. GPx is an antioxidant enzyme which reduces hydrogen peroxide to water in order to limit its harmful effects [43]. GPx was implicated in spatial memory acquisition [44]. In this study, the GPx level was correlated with the at-risk quotient. The higher the level of GPx, the better the performance in reading, writing and other related abilities measured by the dyslexia assessment test. This underscores the potential role of GPx in learning. Additionally, SOD is the major antioxidant defense system against oxides. Consequently, its reduction would induce neuronal damage. It has a protective role against memory decline and it was related to memory deficits when reduced [45]. Furthermore, hippocampal oxidative stress associated with decreased SOD levels has been linked to memory impairment in rats and reduced b cognitive performance in humans [46]. Working memory score reduction has been noticed among participants with LD in the present study. Working memory has been strongly related to learning performance. It is involved in integrating the structures and processes used for temporarily storing and manipulating information, which underscores its role in executive functions [47]. Hence, the low levels of SOD in the participants with LD ± ADHD in this study could have influenced their performance in the used tests and suggests that SOD could participate in modulating the performance of abilities involved in learning. However, neither correlation analysis nor diagnostic value exploration suggested SOD as a possible biomarker for the participants with LD in this study.

To investigate the possible role of KYN in LD, a correlation between its level and the scores of the subtests of the dyslexia assessment test was performed. There was a significant negative correlation between KYN level and scores of one-minute writing subtest. The higher the KYN level, the worse the writing performance. Motor integrity and eye-motor coordination have been linked to writing performance in children with LD [48]. No correlation was noticed with total IQ scores. These findings highlight KYN’s involvement in learning disorder, which occur in the absence of intellectual disability. The present correlation output is in line with previous studies which highlighted the implication of KYN in neurological motor disorders such as temporal lobe epilepsy. These disorders were further associated with cognitive deficits and memory decline [5] which could suggest a relationship between KYN and cognitive dysfunction. Furthermore, some preliminary studies indicated that reducing KYN levels could help individuals with ADHD enhance their cognitive abilities [49].

The evaluated biochemical measures did not differ between the group of children with LD without ADHD and those with LD and coexisting ADHD. The number of children who had ADHD was less than the group without ADHD, and the participants were not recruited from the general population, which could be limitations of the study. Nevertheless, the correlation analysis and the diagnostic utility were not performed for children with LD and comorbid ADHD. They were performed on children who had LD without ADHD. The correlation outputs and the diagnostic utility estimation results in this study suggest that KYN, GPx, and MDA could be promising measures that are linked to the performance of some abilities of children with specific learning disorder or developmental dyslexia. The results of this cross-sectional study suggest that changes observed in the levels of the targeted measures could be considered associations with LD. Therefore, performing future studies investigating the role of these measures as a cause or a result of having LD is recommended. Moreover, implementing other studies investigating the influence of different lines of therapy on these measures is advised.

## Conclusion

Some cognitive abilities differed between the children with LD and those with LD and ADHD, with a significant statistical difference. The presence of coexisting ADHD did not show an influence on the evaluated biochemical measures in children with LD. The KYN system and oxidative stress markers are suggested to be involved in or influence the abilities of children with LD. The mechanism by which these markers can influence cognitive abilities are complicated and could be induced by internal and/or external factors such as stress. The levels of KYN and GPx showed a significant correlation with the writing performance of school-aged children in this study and their at-risk quotients, which represented the skills and abilities involved in learning. Therefore, KYN, MDA, and GPx are proposed as measures to be evaluated and checked for alterations in children with developmental dyslexia or specific learning disorder.

## Data Availability

The datasets used and/or analyzed during the current study are available from the corresponding author on reasonable request.

## Abbreviations

ADHD: Attention deficit hyperactivity disorder
EEG: Electroencephalogram
GPx: Glutathione peroxidase
IQ: Intelligence quotient
KYN: Kynurenine
LD: Learning disorder
LD ± ADHD: LD with or without coexisting ADHD
MDA: Malondialdehyde
MRI: Magnetic resonance imaging
SD: Standard deviation
SOD: Super oxide dismutase
STROBE: Strengthening the reporting of observational studies in epidemiology
NT: Neurotypical

## Footnotes

1 STROBE (Strengthening the reporting of observational studies in epidemiology) is a checklist that is encouraged be used and fulfilled in observational studies such as cross-sectional studies.
